# Ciprofol versus propofol for general anesthesia during thoracoscopic surgery: a randomized double-blind trial

**DOI:** 10.3389/fmed.2026.1923698

**Published:** 2026-10-01

**Authors:** Yu Wang, Yizhi Geng, Yaodong Yan, Xinying Gao, Qi Shi

**Affiliations:** 1 Department of Anesthesiology, Fuyang Normal University Second Affiliated Hospital, Fuyang, Anhui, China; 2 Doctoral Program, Keio University Graduate School of Medicine, Shinjuku-ku, Tokyo, Japan

**Keywords:** bispectral index (BIS), ciprofol, general aneasthesia, heart rate, mean arterial blood pressure (MAP), propofol, thoracoscopic surgery

## Abstract

**Background:**

Ciprofol is a novel intravenous anesthetic with potentially improved hemodynamic stability and fewer injection-related adverse effects than propofol. This study compared ciprofol and propofol for general anesthesia in patients undergoing video-assisted thoracoscopic surgery (VATS).

**Methods:**

In this prospective, randomized, double-blind trial, 120 adults undergoing elective thoracoscopic surgery were randomized to ciprofol (*n* = 60) or propofol (*n* = 60). MAP, HR, and BIS were assessed at eight predefined perioperative time points using linear mixed-effects models. The repeated binary outcome of BIS ≥60 was analyzed using a generalized linear mixed-effects model. Induction characteristics, injection pain, recovery profiles, and perioperative adverse events were also evaluated.

**Results:**

MAP differed significantly between groups, with higher values in the ciprofol group at loss of consciousness and after tracheal intubation. HR was modestly higher at selected early time points, without a significant group-by-time interaction. BIS was higher with ciprofol during the early intraoperative period, and BIS ≥60 was more frequent at loss of consciousness (76.7% vs. 43.3%, *p* < 0.001), but not thereafter. Ciprofol was associated with shorter induction time (38.3 vs. 46.0 s, *p* < 0.001), less injection pain (5.0% vs. 20.0%, *p* = 0.025), and shorter PACU stay (48.0 vs. 55.7 min, *p* = 0.011). No significant differences were observed in supplemental anesthetic requirements, emergence time, or perioperative adverse events.

**Conclusions:**

Ciprofol showed a perioperative profile broadly comparable to propofol, with transient differences in hemodynamic parameters and higher BIS during the early intraoperative period. Ciprofol was associated with faster induction, less injection pain, and shorter PACU stay. Larger multicenter studies are warranted to confirm these findings.

**Clinical Trial Registration:**

https://www.chictr.org.cn/showproj.html?proj=203394, Identifier: ChiCTR2400082924.

## 1 Introduction

Video-assisted thoracoscopic surgery (VATS) is a widely used minimally invasive technique for pulmonary procedures, offering reduced surgical trauma and faster recovery compared with open thoracotomy (1, 2). However, intraoperative management remains challenging due to one-lung ventilation, carbon dioxide insufflation, and airway manipulation with double-lumen endotracheal tubes, all of which may contribute to hemodynamic instability during anesthesia induction and maintenance (3–5).

Propofol is commonly used for intravenous anesthesia because of its rapid onset and favorable recovery profile (6, 7). However, it is associated with dose-dependent hypotension, respiratory depression, and injection pain, which may be particularly relevant in thoracic surgery where stable hemodynamics are essential (6–8).

Ciprofol is a novel intravenous anesthetic and a gamma-aminobutyric acid type A (GABAA) receptor agonist structurally related to propofol (6, 9). It has higher receptor affinity and greater potency, allowing effective anesthesia at lower doses (6, 10). Previous studies suggest that ciprofol may be associated with reduced injection pain and more stable hemodynamics compared with propofol (10–12). However, evidence remains limited in patients undergoing thoracoscopic surgery requiring one-lung ventilation (11, 12).

In addition, the effects of ciprofol on intraoperative anesthetic depth, as assessed by bispectral index (BIS), and its relationship with hemodynamic stability and early postoperative recovery remain insufficiently characterized.

Therefore, we conducted a randomized controlled trial to compare ciprofol and propofol for the induction and maintenance of general anesthesia in patients undergoing thoracoscopic surgery, focusing on hemodynamic stability, anesthetic depth, and postoperative recovery.

## 2 Methods

### 2.1 Study design and ethics

This randomized, double-blind, controlled trial was conducted at the Fuyang Normal University Second Affiliated Hospital. The study was approved by the Institutional Ethics Committee of the hospital (Approval No. [2024]001) and registered at the Chinese Clinical Trial Registry (ChiCTR2400082924). Written informed consent was obtained from all participants. The trial adhered to the Declaration of Helsinki. No external funding or industry support was received.

### 2.2 Participants

Eligible patients were aged 18–75 years, with American Society of Anesthesiologists (ASA) physical status I–III and a body mass index (BMI) of 18.5–27.9 kg/m^2^, scheduled for elective thoracoscopic surgery. Exclusion criteria included neurological or psychiatric disorders (e.g., stroke, epilepsy, dementia, schizophrenia, depression), severe cardiopulmonary or renal dysfunction, known drug allergies, recent exposure to propofol or ciprofol, alcohol or drug abuse within 3 months, and contraindications to study medications. Patients were withdrawn if serious adverse events occurred, protocol violations affected data integrity, anesthesia was inadequate, or withdrawal was requested.

### 2.3 Randomization and blinding

A computer-generated randomization list was used to assign patients to the propofol group (P group) or the ciprofol group (C group). Allocation concealment was maintained using sequentially numbered, opaque, sealed envelopes prepared by an independent statistician.

Double blinding was maintained throughout the study. Anesthesia nurses uninvolved in clinical care prepared study medications in identical syringes. Patients, anesthesiologists, surgeons, nurses, and outcome assessors remained blind until completion of data analysis.

### 2.4 Anesthesia protocol

Standard intraoperative monitoring included electrocardiography, pulse oximetry, noninvasive blood pressure monitoring, end-tidal carbon dioxide, and bispectral index (BIS) (13, 14). Anesthetic induction was initiated with intravenous midazolam (0.04 mg/kg), followed by sufentanil (0.4 μg/kg). The initial loading dose was 2.0 mg/kg for propofol (Group P) and 0.4 mg/kg for ciprofol (Group C), administered intravenously over 30 s. Sedation depth was assessed every 30 s using the Modified Observer's Assessment of Alertness/Sedation (MOAA/S) scale, with loss of consciousness defined as a MOAA/S score ≤1 (15). If the MOAA/S score remained >1 at 2 min after completion of the initial loading dose, a supplemental dose corresponding to 30% of the initial dose (0.6 mg/kg for propofol or 0.12 mg/kg for ciprofol) was administered. Supplemental doses were administered at intervals of at least 1 min, with a maximum of two supplemental doses permitted. If the MOAA/S score remained >1 at 1 min after the second supplemental dose, induction was considered unsuccessful. After loss of consciousness was confirmed (MOAA/S ≤ 1), rocuronium (0.6 mg/kg) was administered intravenously, followed by tracheal intubation.

After loss of consciousness was confirmed and induction was completed, propofol was continuously infused at an initial rate of 6 mg/kg/h, adjustable by 1–2 mg/kg/h according to clinical response within a range of 4–12 mg/kg/h, whereas ciprofol was continuously infused at an initial rate of 0.8 mg/kg/h, adjustable by 0.1–0.4 mg/kg/h within a range of 0.8–2.4 mg/kg/h. Anesthesia was maintained with continuous infusion of propofol or ciprofol, titrated to maintain BIS values between 40 and 60. Additional remifentanil (0.05–0.2 μg/kg/min) and neuromuscular blocking agents were administered as required according to surgical conditions and anesthetic depth. Double-lumen endotracheal intubation was performed after induction of anesthesia, and correct tube placement was confirmed using fiberoptic bronchoscopy. Mechanical ventilation was initiated with a tidal volume of 8–10 mL/kg during two-lung ventilation, while the respiratory rate was adjusted to maintain end-tidal carbon dioxide between 35 and 45 mmHg. After lateral positioning, double-lumen tube placement was reconfirmed, and one-lung ventilation was subsequently established using a tidal volume of 6–8 mL/kg (16, 17). Hemodynamic parameters and oxygenation were continuously monitored throughout the procedure.

Intraoperative hypotension was defined as MAP <65 mmHg lasting for ≥1 min, whereas intraoperative hypertension was defined as an increase in MAP of >20% from baseline lasting for ≥1 min. When hypotension occurred, intravenous methoxamine (3–6 mg) or ephedrine (5–10 mg) was administered as rescue therapy. When hypertension occurred, intravenous esmolol (10–20 mg) was administered as rescue therapy. The same rescue medication protocol was applied to both groups.

### 2.5 Study endpoint

The prespecified primary outcome was longitudinal mean arterial pressure (MAP), assessed at predefined perioperative time points. The primary analysis evaluated the group×time interaction to characterize differences in the temporal pattern of MAP between the ciprofol and propofol groups. Secondary outcomes included heart rate (HR) and bispectral index (BIS) at the same time points, as well as intraoperative variability of MAP, HR, and BIS. Additional secondary endpoints included induction time (from administration of the study drug to loss of consciousness), MOAA/S scores during induction and emergence, requirement for supplemental anesthetic doses during induction, incidence of injection pain, emergence time (from discontinuation of anesthetic infusion to tracheal extubation), and length of stay in the post-anesthesia care unit (PACU). MOAA/S scores during induction were assessed at 30, 60, 90, and 120 s after administration of the study drug, whereas MOAA/S scores during emergence were assessed at 3, 6, 9, and 15 min after discontinuation of anesthetic infusion. Perioperative adverse events were recorded throughout the study period. Injection pain was assessed immediately after administration of the study drug and recorded as present or absent by an independent blinded investigator.

Hemodynamic and anesthetic depth variables, including MAP, HR, and BIS, were recorded at eight predefined perioperative time points: baseline on admission to the operating room (T1), loss of consciousness (T2), after tracheal intubation (T3), skin incision (T4), 5 min after incision (T5), 30 min after incision (T6), 60 min after incision (T7), and at the end of surgery (T8).

Patients were discharged from the PACU when a modified Aldrete score of at least 9 was achieved and routine discharge criteria were fulfilled, including stable hemodynamics, adequate spontaneous ventilation, satisfactory oxygenation, and appropriate recovery of consciousness. PACU discharge decisions were made by anesthesiology staff who were blinded to group allocation.

### 2.6 Sample size calculation

The sample size was determined *a priori* based on the expected group×time interaction for the prespecified primary outcome, longitudinal mean arterial pressure (MAP). Assuming a standardized effect size of *f* = 0.27, a two-sided type I error rate (α) of 0.05, 80% power, eight repeated measurements, and a within-subject correlation of 0.50, approximately 54 patients per group were required. The effect size was specified as a standardized effect size; therefore, no separate absolute standard deviation was used in the sample-size calculation. To allow for approximately 10% potential attrition, protocol deviations, and nonevaluable observations, the target sample size was increased to 60 patients per group, resulting in a total planned enrollment of 120 patients. The calculation was performed using G*Power version 3.1.

### 2.7 Statistical analysis

Statistical analyses were performed using GraphPad Prism version 10.5.0 (GraphPad Software, San Diego, CA, USA). Continuous variables were expressed as mean ± standard deviation (SD), whereas categorical variables were presented as numbers and percentages. Normality of continuous variables was assessed before analysis. Between-group comparisons of continuous variables were performed using the unpaired Student's t test, whereas categorical variables, including perioperative adverse events, were analyzed using the chi-square test or Fisher's exact test, as appropriate.

The longitudinal mean arterial pressure (MAP) analysis was prespecified as the primary analysis, with the group×time interaction representing the primary estimand. MAP was the sole prespecified primary outcome. Serial perioperative measurements, including MAP, heart rate (HR), continuous bispectral index (BIS), and MOAA/S scores, were analyzed using linear mixed-effects models, with group, time, and their interaction as fixed effects and patient as a random effect to account for within-subject correlations. Models were fitted using restricted maximum likelihood (REML). For the primary MAP analysis, a two-sided *α* level of 0.05 was used. For prespecified secondary longitudinal outcomes, including HR, *Δ*MAP, *Δ*HR, BIS, and MOAA/S, the same mixed-effects modeling framework was applied. *Post hoc* pairwise comparisons at individual time points were performed using Šídák adjustment for multiple comparisons, and adjusted *P* values were reported for between-group comparisons at individual time points.

To assess within-patient variability, the standard deviation of serial BIS, MAP, and HR measurements was calculated for each patient and compared between groups using the unpaired t test with Welch’s correction.

The repeated binary outcome of BIS ≥60 was analyzed using a generalized linear mixed-effects model with a binomial distribution and logit link in IBM SPSS Statistics version 27 (IBM Corp., Armonk, NY, USA). Group, time, and the Group×Time interaction were included as fixed effects, with patient specified as the subject to account for repeated measurements within patients. Time was treated as a categorical variable. A diagonal covariance structure was used for the repeated measurements. The significance of the fixed effects was assessed using *F* tests with residual degrees of freedom. Odds ratios (ORs) and 95% confidence intervals (CIs) were calculated from the model estimates.

Mean differences with 95% CIs were calculated where appropriate. No missing data occurred during the study, and all randomized patients were included in the final analysis. Secondary outcomes were not used to determine the overall sample size. All statistical tests were two-sided, and a *P* value < 0.05 was considered statistically significant, except where multiplicity-adjusted *P* values were reported for *post hoc* pairwise comparisons.

## 3 Result

### 3.1 Patient recruitment and baseline characteristics

A total of 125 patients were assessed for eligibility. After exclusions and randomization, 120 patients (60 per group) were included in the final analysis (Figure 1).

**Figure 1:**
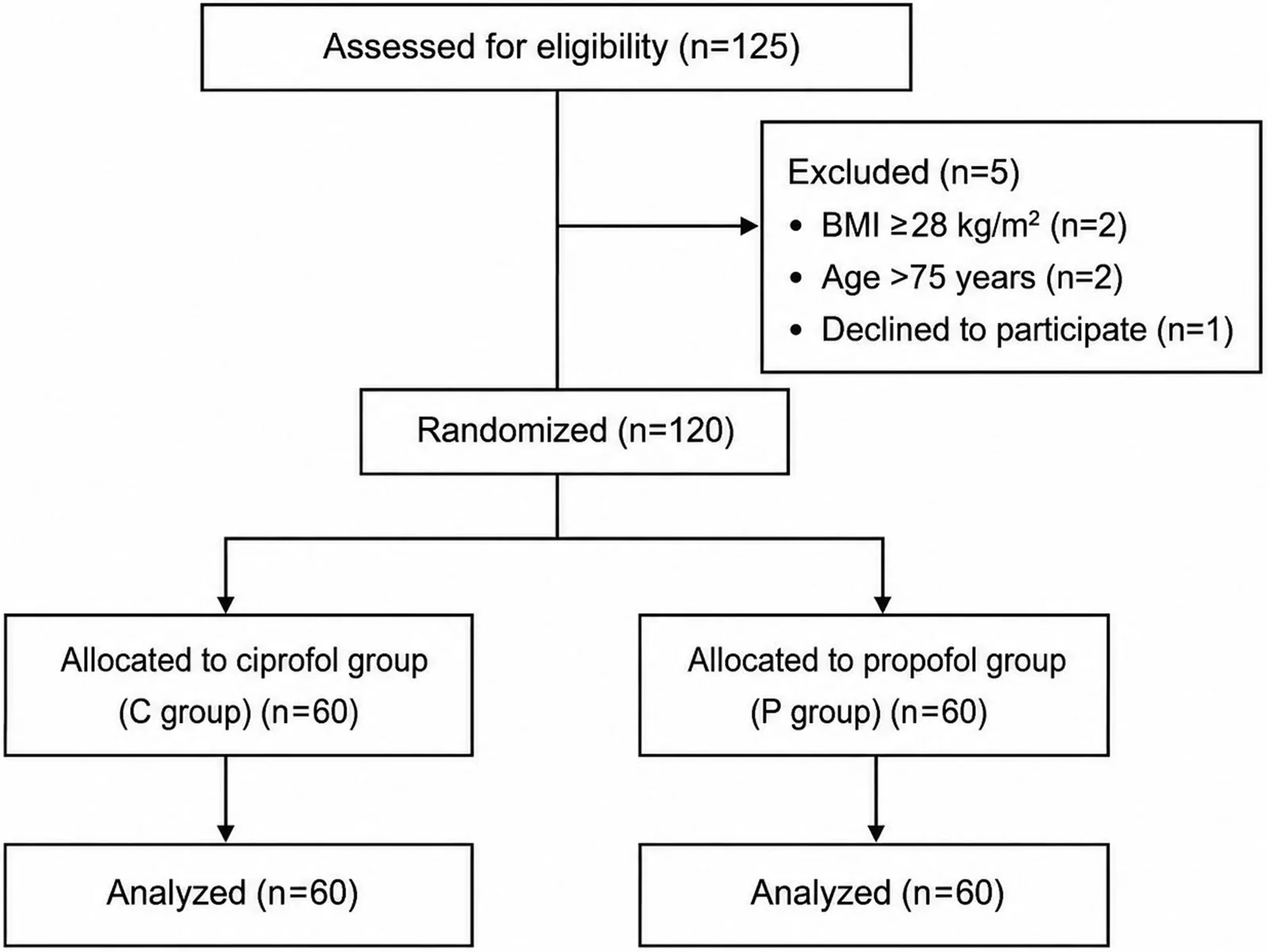
CONSORT flow diagram of study participants.

The patients’ characteristics are shown in (Table 1). There were no significant differences between the two groups in terms of sex, age, weight, height, BMI, ASA, surgical procedure or time of operation and anesthesia.

**Table 1:** Baseline characteristics.

Characteristic	Propofol (*n* = 60)	Ciprofol (*n* = 60)	*p* value
Sex, *n* (%)			0.097
Female	21 (35.0)	31 (51.7)	
Male	39 (65.0)	29 (48.3)	
Age (years)	60.3 ± 12.1	61.7 ± 11.8	0.554
Weight (kg)	64.9 ± 11.3	64.1 ± 12.6	0.704
Height (cm)	166.2 ± 7.8	163.9 ± 7.3	0.091
BMI (kg/m^2^)	23.3 ± 3.4	23.8 ± 3.9	0.507
ASA, *n* (%)			0.732
Ⅰ	10 (16.7)	8 (13.3)	
Ⅱ	41 (68.3)	40 (66.7)	
Ⅲ	9 (15)	12 (20)	
Surgical procedure, *n* (%)			0.421
Wedge resection	18 (30.0)	20 (33.3)	
Segmentectomy	12 (20.0)	10 (16.7)	
Lobectomy	24 (40.0)	23 (38.3)	
Mediastinal surgery	6 (10.0)	7 (11.7)	
Time of operation (min)	129.3 ± 52.0	129.5 ± 55.6	0.989
Time of anesthesia (min)	169.0 ± 56.9	167.6 ± 59.5	0.889

### 3.2 Primary outcome – longitudinal mean arterial pressure

Mean arterial pressure (MAP) showed significant effects of time (F(7, 826) = 115.2, *p* < 0.001), group (F(1, 118) = 4.631, *p* = 0.033), and a significant group×time interaction (F(7, 826) = 3.031, *p* = 0.004), indicating that the between-group differences varied across perioperative time points. Both groups showed an initial decrease in MAP after induction followed by partial recovery during surgery (Figure 2). *Post hoc* analysis demonstrated significantly higher MAP in the ciprofol group at T2 and T3 (adjusted *p* = 0.003 and *p* = 0.019, respectively), with no significant differences at the other time points. The variability of MAP was comparable between the ciprofol and propofol groups (mean SD, 11.13 vs. 12.90; mean difference, −1.77; 95% CI, −3.60 to 0.05; *P* = 0.056). Overall, MAP was higher in the ciprofol group than in the propofol group (mean difference [Ciprofol−Propofol] = 3.46 mmHg, 95% CI 0.28 to 6.64) (Table 2).

**Figure 2:**
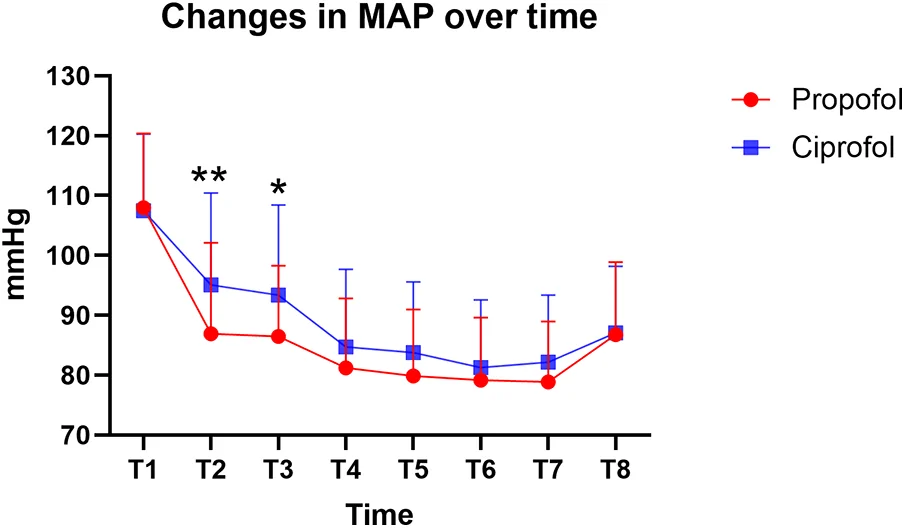
Changes in MAP over time.

**Table 2:** Intraoperative hemodynamic and anesthesia depth variables.

Variable	Propofol (mean)	Ciprofol (mean)	Effect estimate (95% CI)	Interaction *p* value
MAP (mmHg)	85.91	89.37	3.46 (0.28 to 6.64)	0.004
ΔMAP (mmHg)	−24.58	−20.66	3.91 (−0.61 to 8.43)	0.009
HR (bpm)	68.43	71.91	3.48 (0.67 to 6.30)	0.190
ΔHR (bpm)	−3.11	−3.89	−0.78 (−3.44 to 1.89)	0.167
BIS	57.04	59.78	2.74 (1.52 to 3.96)	<0.001

ΔMAP and ΔHR were calculated as the values at each time point minus the baseline value at T1. Effect estimates are expressed as Ciprofol−Propofol.

### 3.3 Secondary hemodynamic outcomes

To further assess the reduction in MAP from baseline, *Δ*MAP was calculated as the MAP at each time point minus the baseline MAP at T1. *Δ*MAP showed a significant effect of time (F(4.041, 476.8) = 29.10, *p* < 0.001) and a significant group×time interaction (F(4.041, 476.8) = 3.437, *p* = 0.009) (Table 2), whereas the overall group effect was not significant (F(1, 118) = 2.941, *p* = 0.089). In Šídák-adjusted *post hoc* comparisons, *Δ*MAP was significantly higher in the ciprofol group at T2 (mean difference [Ciprofol−Propofol] = 8.65 mmHg, 95% CI 2.29 to 15.01; adjusted *p* = 0.002), with no significant between-group differences at the other time points.

Heart rate (HR) showed significant effects of time (F(4.098, 483.6) = 8.058, *p* < 0.001) and group (F(1, 118) = 5.999, *p* = 0.016), but not of the group×time interaction (F(4.098, 483.6) = 1.535, *p* = 0.190), indicating that the between-group difference in HR remained relatively consistent over time. HR followed a similar perioperative pattern in both groups, with no significant difference in HR variability (*p* = 0.6544) (Figure 3). *Post hoc* analysis showed significantly higher HR in the ciprofol group at T3 and T5 (adjusted *p* = 0.021 and *p* = 0.042, respectively), whereas no significant differences were observed at the other time points. The variability of HR was comparable between the ciprofol and propofol groups (mean SD, 5.40 vs. 5.71; mean difference, −0.30; 95% CI, −1.64 to 1.03; *P* = 0.654). Overall, HR was higher in the ciprofol group than in the propofol group (mean difference [Ciprofol−Propofol] = 3.48 bpm, 95% CI 0.67 to 6.30) (Table 2).

**Figure 3:**
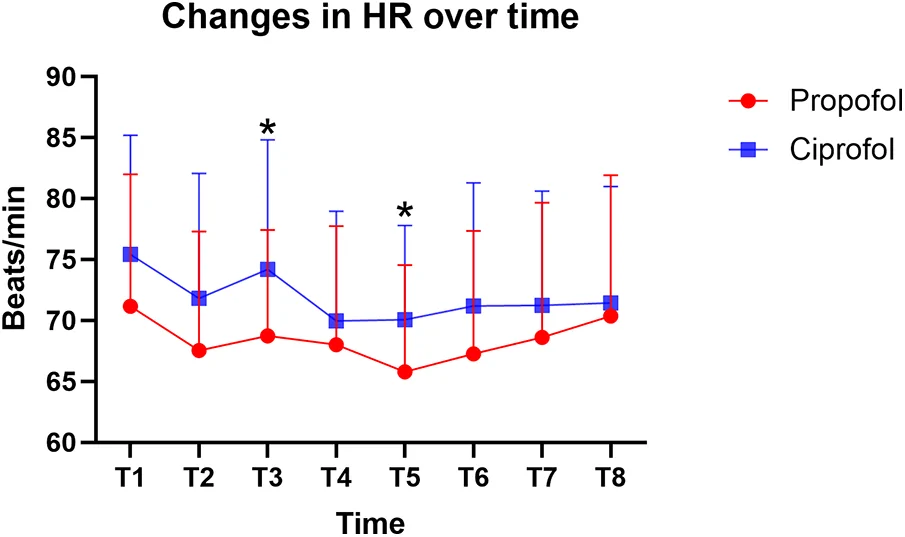
Changes in HR over time.

Analysis of *Δ*HR showed no significant group×time interaction (F(3.785,446.6) = 1.639, *p* = 0.167) (Table 2), and no significant between-group differences were observed at any individual time point after Šídák adjustment (all adjusted *p* > 0.05).

### 3.4 Secondary outcome – anesthetic depth (BIS)

Bispectral index (BIS) showed significant effects of time, group, and their interaction (all *p* < 0.001), indicating that between-group differences varied over time. *Post hoc* analysis demonstrated higher BIS values in the ciprofol group at T2–T6 (all adjusted *p* < 0.01), whereas no significant differences were observed at T1, T7, or T8 (Figure 4). The BIS standard deviation was lower in the ciprofol group than in the propofol group (15.50 vs. 16.46; difference, −0.96; 95% CI, −1.71 to −0.21; *p* = 0.013). Overall, BIS values were higher in the ciprofol group than in the propofol group (59.78 vs. 57.04), with a between-group mean difference of 2.74 BIS units (95% CI, 1.52–3.96; *p* < 0.001), which was consistent with the observed group means (Table 2).

**Figure 4:**
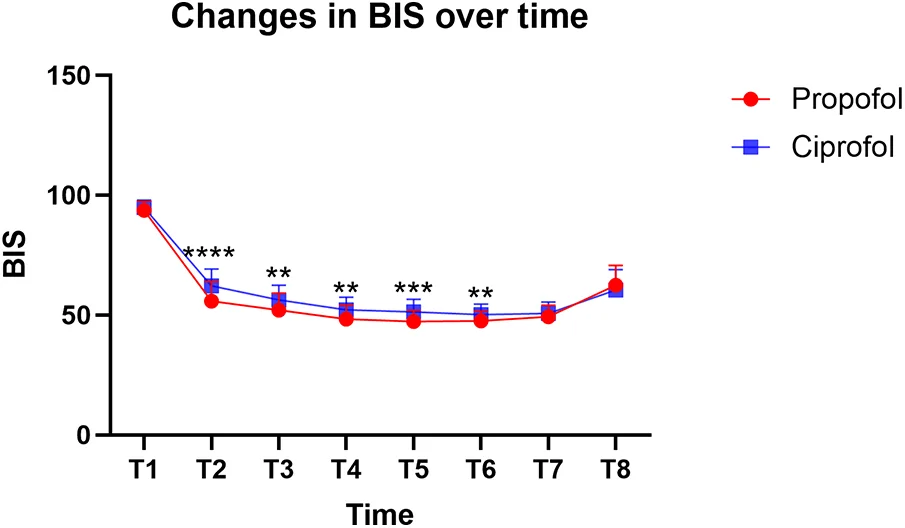
Changes in biS over time.

A significant group×time interaction was observed for the incidence of BIS ≥60 (F(7, 944) = 4.562, *p* < 0.001). At T2, the incidence of BIS ≥60 was significantly higher in the ciprofol group than in the propofol group (76.7% vs. 43.3%; OR, 4.429; 95% CI, 2.053–10.042; *p* < 0.001). No significant between-group differences were observed at T3–T8 (all *p* > 0.05) (Table 3).

**Table 3:** Incidence of biS ≥ 60 at each perioperative time point.

Time point	Propofol, *n* (%)	Ciprofol, n(%)	*p* value
T1	60 (100.0)	60 (100.0)	>0.999
T2	26 (43.3)	46 (76.7)	<0.001
T3	9 (15.0)	16 (26.7)	0.057
T4	1 (1.7)	0 (0.0)	0.279
T5	0 (0.0)	1 (1.7)	0.720
T6	0 (0.0)	0 (0.0)	0.493
T7	1 (1.7)	0 (0.0)	0.273
T8	38 (63.3)	33 (55.0)	0.170

### 3.5 Secondary outcomes – induction, emergence, and recovery

Induction time was shorter in the ciprofol group than in the propofol group (38.3 vs. 46.0 s, *p* < 0.001), whereas the proportion of patients requiring additional anesthetic doses was similar between groups (11.7% vs. 3.3%, *p* = 0.163), and no induction failure was observed in either group (Table 4).

**Table 4:** Induction & emergence.

Outcome	Propofol	Ciprofol	Effect estimate (95% CI)	*p* value
Induction time (s)	46.03	38.30	MD −7.73 (−11.59 to −3.88)	<0.001
Additional dose, *n* (%)	2 (3.3)	7 (11.7)	OR 3.84 (0.75–19.6)	0.163
Induction MOAA/S, group×time interaction	—	—	—	0.024
Emergence time (min)	14.22	16.10	MD 1.88 (−0.80 to 4.56)	0.169
Emergence MOAA/S, group×time interaction	—	—	—	0.034
PACU stay (min)	55.72	47.97	MD −7.75 (−13.70 to −1.80)	0.011

MD, Ciprofol−Propofol; OR, Ciprofol vs Propofol.

MOAA/S scores during induction showed a significant group×time interaction (F(1.397, 164.8) = 4.445, *p* = 0.024) (Table 4). *Post hoc* analysis revealed a significant between-group difference at 60 s after induction (adjusted *p* = 0.0003), whereas no significant differences were observed at 30, 90, or 120 s.

MOAA/S scores during emergence showed no significant group effect (F(1, 118) = 1.175, *p* = 0.281), although a significant group×time interaction was observed (F(2.527, 298.2) = 3.118, *p* = 0.034) (Table 4). However, *post hoc* comparisons demonstrated no significant between-group differences at any assessment time point (3, 6, 9, or 15 min) after adjustment for multiple comparisons.

Emergence time did not differ significantly between groups (14.22 vs. 16.10 min, *p* = 0.169), postoperative recovery in the post-anesthesia care unit (PACU) was shorter in the ciprofol group compared with the propofol group (47.97 vs. 55.72 min, *p* = 0.011) (Table 4).

### 3.6 Secondary outcomes – safety

The incidence of injection pain was significantly lower in the ciprofol group than in the propofol group (5.0% vs. 20.0%, *p* = 0.025). No significant differences were observed between groups in the incidence of perioperative hypotension (16.7% vs. 18.3%, *p* > 0.999), hypertension (11.7% vs. 8.3%, *p* = 0.762), bradycardia (25.0% vs. 21.7%, *p* = 0.829), tachycardia (13.3% vs. 20.0%, *p* = 0.463), or myoclonus (1.7% vs. 0%, *p* > 0.999). (Table 5).

**Table 5:** Perioperative safety outcomes.

Perioperative safety outcomes	Propofol, *n* (%)	Ciprofol, n(%)	*p* value
Injection pain	12 (20.0)	3 (5.0)	0.025
Hypotension	11 (18.3)	10 (16.7)	>0.999
Hypertension	5 (8.3)	7 (11.7)	0.762
Bradycardia	13 (21.7)	15 (25.0)	0.829
Tachycardia	12 (20.0)	8 (13.3)	0.463
Myoclonus	0 (0.0)	1 (1.7)	>0.999

## 4 Discussion

In this randomized controlled trial, ciprofol demonstrated intraoperative hemodynamic profiles broadly comparable to those of propofol during thoracoscopic surgery. Although BIS values were higher during the early intraoperative period with ciprofol, no increase in supplemental anesthetic requirements was observed, and no clinically apparent signs of inadequate anesthesia were recorded. In addition, ciprofol was associated with a shorter induction time, a lower incidence of injection pain, and a shorter PACU stay. Both anesthetic regimens demonstrated favorable perioperative safety profiles, with no severe adverse events observed.

Thoracoscopic surgery presents unique anesthetic challenges because one-lung ventilation, artificial pneumothorax, positive-pressure ventilation, and the use of double-lumen endotracheal tubes may contribute to substantial perioperative hemodynamic fluctuations (18, 19). Therefore, maintaining cardiovascular stability is an important goal during thoracic anesthesia. In the present study, both groups demonstrated similar perioperative MAP and HR trajectories, characterized by an initial decline after induction followed by gradual stabilization during surgery. Although statistically significant differences were observed at several time points, the absolute magnitude of these differences remained modest. The between-group differences in MAP and HR were approximately 3–4 mmHg and 3–4 beats/min, respectively, at the time points showing significant differences, and no significant differences in variability were identified. The significant between-group differences observed at T2 and T3 likely reflect transient hemodynamic responses during the induction and tracheal intubation phases of anesthesia, with T2 corresponding to loss of consciousness and T3 to the immediate post-intubation period. Importantly, these differences were most pronounced during the early induction and intubation periods and were not sustained at subsequent intraoperative time points. Although a significant group×time interaction was observed for MAP, the between-group differences were confined primarily to T2 and T3. Thus, while these differences were statistically significant, their small absolute magnitude limits their interpretation as evidence of a clinically meaningful hemodynamic benefit. In contrast, no significant group×time interaction was observed for HR, indicating that the between-group difference in HR remained relatively consistent over time.

Previous studies have shown that ciprofol exhibits anesthetic efficacy similar to propofol in procedural sedation and general anesthesia (20–22). Some reports have suggested that ciprofol may be associated with less cardiovascular depression and a lower incidence of hypotension than propofol. However, findings across studies remain inconsistent, likely due to differences in surgical procedures, anesthetic regimens, and perioperative management strategies (23–25). Our results support the view that ciprofol and propofol demonstrate broadly similar hemodynamic behavior during thoracoscopic surgery, with no significant difference in the incidence of clinically defined perioperative hemodynamic adverse events (22, 26). Although MAP and HR were slightly higher in the ciprofol group during the early anesthesia period, the absolute differences were small and their clinical significance remains uncertain. These findings therefore should be interpreted as differences in the perioperative hemodynamic profile rather than evidence of a clinically meaningful hemodynamic advantage of ciprofol.

A notable finding of this study was the higher BIS values observed in the ciprofol group during the early intraoperative period. Although BIS values were higher during the early intraoperative period, no increase in supplemental anesthetic requirements was observed. Emergence profiles and MOAA/S scores were also comparable between groups, suggesting that the observed BIS differences were not associated with clinically relevant differences in anesthetic depth.

Previous randomized studies comparing ciprofol with propofol have generally reported comparable or lower BIS values with ciprofol during general anesthesia, together with reduced hemodynamic suppression (27). In contrast, our findings demonstrated higher BIS values during the induction and tracheal intubation period. This may reflect transient physiological responses to anesthetic induction and airway stimulation, as well as differences in pharmacodynamic onset and EEG sensitivity during early anesthesia.

There was no significant difference in baseline BIS values between the two groups at T1. Although BIS values were higher in the ciprofol group from T2 to T6, a significant group×time interaction was observed for the incidence of BIS ≥60. This interaction was primarily driven by the higher incidence of BIS ≥60 in the ciprofol group at T2 (76.7% vs. 43.3%), whereas no significant between-group differences were observed from T3 to T8. Thus, although ciprofol was associated with slightly higher BIS values during the early intraoperative period, the difference in the proportion of patients with BIS ≥60 was transient and was not observed during the subsequent maintenance period. The lower BIS variability observed with ciprofol may reflect differences in EEG signal dynamics rather than clinically meaningful differences in anesthetic depth. Further studies are warranted to clarify the relationship between ciprofol and BIS-derived indices.

In addition to comparable intraoperative stability, ciprofol demonstrated several perioperative advantages. Induction time was shorter in the ciprofol group, indicating more rapid achievement of the predefined clinical endpoint of loss of consciousness (28–30). Furthermore, injection pain occurred less frequently with ciprofol than with propofol (29, 30). Injection pain is a common adverse effect of propofol and may negatively influence patient comfort and perioperative experience (31, 32). The reduced incidence of injection pain observed with ciprofol may be related to differences in emulsion formulation and aqueous-phase drug concentration between the two agents (32, 33).

Another clinically relevant finding was the shorter PACU stay observed in the ciprofol group. Enhanced recovery after surgery (ERAS) protocols are increasingly emphasized in thoracic surgery, where rapid postoperative recovery and efficient perioperative management are important clinical goals (34–36). Although emergence time itself was comparable between groups, the reduction in PACU stay may indicate improved early postoperative recovery efficiency following ciprofol anesthesia. Additional studies are needed to determine whether this finding translates into broader postoperative recovery benefits.

Our study differs from the previous randomized controlled trial of ciprofol versus propofol in thoracoscopic pulmonary surgery (30) in several important respects. Although the study populations were broadly comparable, as both studies enrolled patients undergoing thoracoscopic pulmonary surgery, the present study included twice as many participants (120 vs. 60). More importantly, the anesthetic protocols differed in several respects. The previous study used ciprofol 0.4 mg/kg with infusion-based anesthesia, target-controlled infusion of propofol for induction and maintenance, and concomitant remimazolam throughout surgery. In contrast, our study used fixed-dose bolus induction with ciprofol (0.4 mg/kg) or propofol (2.0 mg/kg), with loss of consciousness defined by serial MOAA/S assessment and supplemental anesthetic dosing permitted according to predefined criteria. Maintenance hypnotic anesthesia was provided with ciprofol or propofol and titrated to maintain BIS values between 40 and 60, without concomitant remimazolam. These differences in anesthetic protocols are relevant when interpreting the hemodynamic and BIS findings, as the observed responses may reflect the overall anesthetic regimen rather than the study drug alone.

The outcome framework also differed between the two studies. The previous study focused on perioperative cardiovascular events and selected time-point changes in MAP and HR, whereas our study prespecified longitudinal MAP as the primary outcome and formally evaluated group, time, and group×time effects across predefined perioperative time points. This longitudinal approach characterized the temporal pattern of hemodynamic responses rather than relying primarily on isolated time-point comparisons. Although the absolute differences in MAP and HR were modest and were not accompanied by differences in the incidence of clinically defined hemodynamic adverse events, this analysis identified a distinct temporal pattern of blood pressure response between the two anesthetic regimens. The present study also provided clinically relevant information on anesthetic depth and clinical responsiveness that was not identified in the previous study. Whereas the previous study found no significant between-group differences in BIS at any assessed time point, our study identified a significant group×time interaction, with higher BIS values in the ciprofol group from T2 to T6. The incidence of BIS ≥60 was also higher with ciprofol at T2, while BIS variability was lower. Importantly, these differences were not accompanied by a greater requirement for supplemental anesthetic doses or clinically apparent signs of inadequate anesthesia. Thus, the BIS findings indicate that ciprofol may produce a different BIS profile from propofol despite achieving comparable clinical control of anesthesia, an observation that is relevant to the interpretation of BIS during ciprofol anesthesia.

Serial MOAA/S assessment provided a more detailed characterization of the time course of clinical responsiveness during induction. In our study, the MOAA/S trajectory showed a significant group×time interaction, with a significant between-group difference at 60 s after induction (adjusted *p* = 0.0003), whereas no significant differences were observed at 30, 90, or 120 s. Thus, compared with the previous study, which assessed induction primarily by loss of the eyelash reflex, our findings indicate that the difference between ciprofol and propofol in clinical responsiveness was most apparent during the early induction phase rather than being uniformly present throughout induction. During emergence, although the overall MOAA/S trajectories showed a significant group×time interaction, no individual *post hoc* comparison remained significant after adjustment, indicating that the observed difference in the overall recovery trajectory did not translate into a statistically significant between-group difference at any specific assessment time point.

Finally, PACU stay was evaluated as an early postoperative recovery outcome that was not assessed in the previous study. The shorter PACU stay observed in the ciprofol group provides clinically relevant information on early postoperative recovery, although PACU duration is influenced by multiple perioperative factors and should therefore not be interpreted as evidence of superior overall postoperative recovery.

Thus, the novelty of the present study does not lie in the initial comparison of ciprofol and propofol during thoracoscopic surgery, which has already been reported (30). Rather, the present study addresses complementary clinical questions by providing a longitudinal evaluation of hemodynamic responses and integrating these findings with BIS-derived hypnotic monitoring, serial assessment of clinical responsiveness using MOAA/S, and an early postoperative recovery outcome. The combination of these outcomes provides additional information on the temporal hemodynamic response, BIS characteristics, early clinical responsiveness, and immediate postoperative recovery associated with ciprofol compared with propofol, thereby complementing the cardiovascular event-focused assessment reported previously.

In addition to the lower incidence of injection pain, no significant differences were observed between groups in the incidence of perioperative hypotension, hypertension, bradycardia, tachycardia, or myoclonus. Although the present study was not powered to detect differences in uncommon adverse events, the incidence of clinically defined hemodynamic adverse events was similar between groups. The relatively small absolute differences in MAP and HR, together with the similar incidence of clinically defined hemodynamic adverse events, suggest broadly comparable hemodynamic profiles between the two groups. Notably, one patient in the ciprofol group experienced myoclonus during induction, whereas no cases were observed in the propofol group. Although the incidence was low and no statistically significant difference was detected, previous studies have reported occasional myoclonus or muscle fasciculation following ciprofol administration, particularly during anesthetic induction, suggesting that this phenomenon may represent a characteristic but uncommon adverse effect of ciprofol. Further investigation is warranted to clarify its underlying mechanism and clinical significance (37).

Several limitations of this study should be acknowledged. First, this was a single-center study with a relatively limited sample size, which may restrict the generalizability of the findings. Second, BIS was used as a surrogate indicator of anesthetic depth and may not fully reflect cortical awareness or anesthetic adequacy. Third, long-term postoperative outcomes, including postoperative cognitive recovery and patient-reported outcomes, were not evaluated. Only patients undergoing thoracoscopic surgery were included; therefore, caution is warranted when extrapolating these findings to other surgical populations. Finally, although MAP <65 mmHg and changes in MAP from baseline were evaluated, the duration and cumulative burden of hypotension, number of discrete hypotensive episodes, and cumulative vasopressor requirements were not predefined as study outcomes and were not prospectively collected using standardized criteria. These measures could therefore not be reliably reconstructed retrospectively. Consequently, the present study cannot determine whether the observed differences in MAP translated into differences in the duration, frequency, or cumulative clinical burden of hypotension. Further prospective studies incorporating standardized assessment of these outcomes are warranted.

## 5 Conclusions

In patients undergoing thoracoscopic surgery, ciprofol was associated with a lower incidence of injection pain, shorter induction time, and shorter post-anesthesia care unit (PACU) stay compared with propofol. Although BIS values were higher during the early intraoperative period in the ciprofol group, the two anesthetic regimens demonstrated broadly comparable overall perioperative hemodynamic profiles, with only transient between-group differences during the early induction and intubation periods. Importantly, the absolute differences in MAP and HR were small and were not accompanied by differences in the incidence of clinically defined hemodynamic adverse events. As a single-center randomized study, these findings suggest that ciprofol may provide a feasible alternative to propofol for anesthesia in thoracoscopic surgery, while further multicenter studies are warranted to confirm these findings and evaluate their clinical implications.

## Data Availability

The raw data supporting the conclusions of this article will be made available by the authors, without undue reservation.
